# Is RAGE the receptor for inflammaging?

**DOI:** 10.18632/aging.102256

**Published:** 2019-09-08

**Authors:** Marie Frimat, Thibault Teissier, Eric Boulanger

**Affiliations:** 1 University of Lille, INSERM, CHU Lille, U995 - Lille Inflammation Research International Center, F-59000 Lille, France; 2 CHU Lille, Department of Nephrology, F-59000 Lille, France; 3 CHU Lille, Department of Geriatrics, F-59000 Lille, France

**Keywords:** aging, inflammaging, pattern recognition receptor (PPR), receptor for advanced glycation end-products (RAGE)

In its full-length (FL) form the receptor for advanced glycation end products, RAGE, is a multi-ligand, transmembrane receptor promoting activation of key pro‐inflammatory and pro‐oxidative pathways. The deleterious effects of its activation *via* the binding of AGEs (the advanced glycation end products after which it is named) are widely reported, especially in diabetes mellitus. Indeed, our current understanding of RAGE relies heavily upon research on this metabolic disorder, but it is simplistic to apprehend this receptor solely within a diabetic context or through its interactions with AGEs. RAGE is more broadly implicated in both immunity and inflammation: it belongs to the immunoglobulin superfamily and its locus is found in the major histocompatibility complex Class III; more than 28 RAGE ligands are known, many of which are damage-associated molecular patterns (DAMPs) or pathogen-associated molecular patterns (PAMPs); cellular responses to RAGE activation are multifarious and involve the expression of pro-inflammatory, -oxidative, -adhesion, -apoptotic, -angiogenic and pro-fibrotic molecules.

RAGE can thus be more accurately considered a pattern recognition receptor (PRR), and has been labelled a “noncanonical Toll-like receptor (TLR)” by some authors [1]. This wider involvement of RAGE signalling nevertheless remains poorly-studied relative to research involving diabetes and AGEs, but evidence is accumulating of its role in what has come to be known as “inflammaging” [2]. RAGE deletion has been shown to be protective against both cardiovascular and Alzheimer’s diseases in RAGE^-/-^ mice, and while the impact of anti-RAGE therapeutics remains to be demonstrated in humans, laboratory results highlight the potential of targeting this receptor to address multiple public health issues. In addition, we recently reported a significant prevention of aged-related nephrosclerosis lesions in RAGE^-/-^ mice, suggesting RAGE also plays a crucial role in renal aging [3] and raising the question as to whether RAGE signalling could be a driver of “physiological” aging.

RAGE has obvious similarities with other PRRs and there are acknowledged pro-aging mechanisms such as oxidative stress, mitochondrial dysfunction or inflammasome activation resulting from its interaction with several of its ligands. The concomitant, age-related increase of circulating DAMPs (*cf.* the “Garb-aging” theory [4]) and the expression of RAGE on many cell membranes, even in the absence of a pathological event, could favour low-grade, persistent, pro-inflammatory processes which in turn could drive increased production of DAMPs and expression of RAGE. This pro-aging vicious circle of events places RAGE firmly in the spotlight as a key-actor in inﬂammaging, not least because senescent cells also produce RAGE ligands like HMGB1 and S100s. This hypothesis is attractive and opens up significant possibilities in the development of anti-RAGE therapeutics, but many questions remain. To what extent do the different RAGE ligands compete for binding, and how does this competition modulate its activation? Are the activated signalling pathways ligand-specific, or perhaps specific to the configuration of RAGE in its homodimeric, oligomeric or proposed heterodimeric (*eg.* with TLRs) forms? Are there negative effects to RAGE inhibition?

Indeed, the physiological roles of RAGE remain to be fully elucidated. Its depletion is not lethal and RAGE knockout mice develop normally without evident defects or reduced fertility. In human adults, its expression becomes weak in most tissues at a basal state. However, RAGE is conserved across mammal species and its high constitutive and ubiquitous expression in embryonic life suggests it might be important in early development. Interestingly, it has recently been cited as a key player in the immunometabolic networks controlling responses to nutrient supply in a mouse model of adipocyte-speciﬁc RAGE deletion [5]. The authors proposed that RAGE’s primal endogenous function could belong to the “thrifty genotype”, and that its innate mechanism is the suppression of adipocyte metabolism and energy expenditure, perhaps partly explaining its preservation through evolution.

Further, most detrimental effects of RAGE stimulation are prominent mostly during post-reproductive life, but some isoforms do not have an entirely negative impact: nuclear RAGE has been implicated in DNA repair [6], while both the endogenous secretory RAGE (esRAGE) and the cleaved RAGE (cRAGE) – two forms of soluble RAGE (sRAGE) – can serve as a decoy for RAGE-ligands and/or interact with FL-RAGE to block its intracellular signalling. Notably, an age-related decrease of circulating cRAGE has been reported in healthy humans which was inversely correlated with inflammatory markers [7]. This could indicate a reduced capacity with age to bind ligands such as DAMPs, thus favouring inflammaging and age-related diseases. A murine model overexpressing circulating sRAGE has recently been generated [8] and may help to investigate the relationship between different RAGE isoforms and inﬂammaging.

The driving mechanisms of aging remain to be fully elucidated. Multiple theories have been proposed which more or less support the thesis of a programmed process – it seems likely that no single factor is responsible, but rather a combination of factors, occurring in an adaptive manner, defines individual aging. We are firmly of the opinion RAGE is involved in the course of aging. With its dynamically-regulated expression and multi-faceted role throughout an organism’s lifespan, it could well be included in antagonistic pleiotropy theories. Perhaps RAGE could even be more generally considered as a receptor that participates in selection for healthy aging. RAGE’s functions are undeniably and fascinatingly complex – much work remains to decipher its role in aging, to provide a more complete vision of this receptor’s role in healthspan, and to understand how to advantageously modulate its activation for successful aging.
